# Vitamin K protects against lipopolysaccharide-induced intestinal inflammation in piglets by inhibiting the MAPK and PI3K-AKT pathways

**DOI:** 10.3389/fnut.2025.1704168

**Published:** 2025-11-17

**Authors:** Huakai Wang, Ruiyu Ma, Renrong Qi, Zhen Liu, Yinghao Li, Xudong Wu, Chunfang Zhao, Qiugang Ma, Kai Zhan

**Affiliations:** 1 Anhui Provincial Key Laboratory of Livestock and Poultry Product Safety, Institute of Animal Husbandry and Veterinary Medicine, Anhui Academy of Agricultural Sciences, Hefei, China; 2 College of Animal Science, Anhui Science and Technology University, Chuzhou, China; 3 Department of Animal Nutrition and Feed Science, College of Animal Science and Technology, China Agricultural University, Beijing, China

**Keywords:** Vitamin K, intestinal inflammation, tight junction protein, piglets, IPEC-J2 cells

## Abstract

**Background:**

The benefits of Vitamin K (VK) in mitigating inflammation have been well-established, while the underlying mechanisms remain not yet fully elucidated. This study aimed to investigate its protective effects and underlying mechanisms in LPS-induced intestinal inflammation of piglets.

**Methods:**

In a 21-day study, 24 weaned piglets were randomly assigned to 4 groups: CON group (basal diet), VK group (basal diet + 4.5 mg/kg VK3), LPS group (basal diet + LPS challenge), LPS+VK (basal diet + 4.5 mg/kg VK3 + LPS challenge). On day 21, LPS or saline was administered to the piglets by intraperitoneal injection. The IPEC-J2 cells were treated with or without VK and LPS for 24 h and analyzed with various assays.

**Results:**

Morphological analysis revealed that VK significantly restored the decreased villus height and ratio of villus height to crypt depth induced by LPS. VK effectively upregulated tight junction proteins claudin-1 expression. Furthermore, VK notably suppressed the overexpression of TNF-α, IL-1β, IL-6, and IL-8, as well as the concentrations of diamine oxidase (DAO) and reactive oxygen (ROS), while restoring the reduced expression of *glutathione peroxidase* (*GSH-Px*) and *superoxide dismutase (SOD)*. The combined analysis of transcriptome and proteome, as well as the Western blot verification, indicated that VK mainly mediates the restriction of the MAPK and PI3K-AKT signaling pathways. Moreover, VK relieved gut microbiota dysbiosis, such as decreasing in *Spirochaetato*.

**Conclusion:**

These results indicated that VK alleviated intestinal inflammation in piglets by inhibiting the MAPK and PI3K-AKT signaling pathways as well as the regulation of in the gut microbiota.

## 1 Introduction

The intestine stands as the largest immune organ in the body, essential not only for digesting and absorbing nutrients but also for effectively defending against pathogenic microorganisms (1, 2). Weaned piglets are highly predisposed to intestinal dysfunction due to stressors including environmental/dietary changes, immature digestive tracts, and compromised immunity, leading to diarrhea, growth retardation, elevated morbidity and mortality, causing substantial economic losses in the swine industry (3, 4). Moreover, the shift toward antibiotic-free feed policies has exacerbated the challenge of intestinal immune dysregulation caused by uncontrolled pathogens, posing a serious threat to the sustainable development of swine farming (5). Thus, it is vital to develop safe and efficient strategies aimed at preserving intestinal integrity for animal health and the optimization of swine farming.

Recently, interest in natural nutritional supplements has grown due to their potential to support intestinal barrier function and alleviate symptoms of gut disorders (6, 7). Vitamin K (VK) is a crucial micronutrient made up of naphthalene quinone compounds with isoprene-type side chains. In addition to its roles in blood clotting and bone health (8–10). VK is increasingly gaining attention for its effects on intestinal health. It has been reported that VK can enhance the activity of antioxidant enzymes such as superoxide dismutase (SOD), catalase (CAT), glutathione peroxidase (GSH-Px), glutathione S-transferase (GST), and glutathione reductase (GSR) in the liver, pancreas, and intestines of juvenile carp, thereby exerting its antioxidant function (11). Moreover, a deficiency of VK would aggravate DSS-induced colitis in mice, while supplementing with VK reduces pro-inflammatory cytokine production and improves both shortened colon length and weight loss (12). *In vitro* studies show that VK effectively reduces the activation of nuclear factor kappa B (NF-κB) and inhibits IKKα/β phosphorylation, thereby suppressing inflammation in human monocyte-macrophage THP-1 cells and mouse monocyte-macrophage cells induced by lipopolysaccharide (LPS) (13). Additionally, VK can inhibit inflammation by inhibiting the production of IL-6 in human macrophages and fibroblasts (14). Although these previous studies have provided evidence for the anti-inflammatory and antioxidant functions of VK, the impact of VK on the intestinal health of weaned piglets and its underlying mechanism remain unclear.

Therefore, this study attempted to investigate the protective regulatory role of VK and its mechanisms on the intestinal health by using the LPS-challenged model *in vivo* and *in vitro*, aiming to provide a theoretical basis for the potential administration of VK in regulating the intestinal health of weaned piglets.

## 2 Materials and methods

### 2.1 Animals and experiment design

This study was performed at Animal Experiment Base of Anhui Academy of Agricultural Sciences (Hefei, China). All animal experimental procedures were in accordance with the Chinese Guidelines for Animal Welfare and Experimental Protocols and were approved by the Anhui Academy of Agriculture Science Ethics Committee (AAAS2025-12).

Twenty-four piglets (Duroc × Landrace × Yorkshire) aged 28 d with an average initial body weight of 7.54 ± 0.74 kg were randomly allocated into four dietary treatment groups (CON, VK, LPS, and LPS+VK groups) based on their body weight and gender, with six replicates per group and one piglet per replicate. Piglets in the CON and LPS groups were given a corn-soybean meal basal diet, and VK and LPS+VK group piglets were given a corn-soybean meal basal diet that was supplemented with 4.5 mg/kg VK3. The basal diet met the nutritional recommendations proposed by the National Research Council for piglets and nutrient levels are shown in Table 1 (15). On day 21, LPS and LPS+VK groups were injected intraperitoneally with LPS (100 μg/kg body weight; *E. coli* serotype 055:B5, Sigma) to mimic continued intestinal damage based on previous studies (16, 17). The piglets in the CON and VK groups were injected with the equal amount of saline. Each piglet was nursed in a single cage with a single-side feeder and nipple drinker. All the weaned piglets were housed in a room with the temperature (25–30 °C) and humidity (60% ± 10%). All piglets had unrestricted access to feed and water, and the experiment lasted for 21 day.

**Table 1:** Composition and nutrient level of basal diet.

**Ingredient**	**Contents**	**Nutrient level^b^**	**Calculated value**
Corn	65.06	Digestible Energyb, MJ/kg	16.20
Soybean meal 46%	14.00	Crude protein, %	19.31
Extruded soybean	6.00	Calcium, %	0.81
Soy protein concentrate	4.00	Available Phosphorus, %	0.40
Fish meal	2.00	Lysine, %	1.51
Whey powder	5.00	Methionine, %	0.43
Calcium hydrogen phosphate	1.15	Threonine, %	0.92
Limestone	1.00	Tryptophan, %	0.26
Salt	0.30		
Lysine 98.5%	0.61		
Methionine 98.5%	0.12		
Threonine	0.20		
Tryptophan	0.06		
Premix^a^	0.50		
Total	100		

^a^Supplied for each kilogram of the diet: vitamin A, 10,000 IU; vitamin D_3_, 400 lU; vitamin E, 10 mg; vitamin K_3_, 0.5 mg; vitamin B_1_, 0.5 mg; vitamin B_2_, 3.75 mg; vitamin B_6_, 2 mg; vitamin B_12_, 24 μg; biotin, 0.3 mg; folic acid, 3 mg; nicotinic acid, 8.75 mg; pantothenic acid, 6.25 mg; choline, 0.4 g; Fe, 96 mg; Cu, 8 mg; Mn, 40 mg; Zn, 120 mg; I, 0.3 mg; Se, 0.25 mg.

^b^Calculated value.

### 2.2 Growth performance and sample collection

Piglets were weighted individually on the first and last day of the experiment. Feed consumption was recorded for each piglet. Growth performance was evaluated based on the average daily gain (ADG), average daily feed intake (ADFI), and ADFI/ADG. Four hour after administering LPS or saline, blood was collected via anterior vena cava and serum was obtained through centrifugation at 3,000 × *g* and 4 °C for 15 min. Then, the serum samples were stored at −80 °C for subsequent analysis. After the collection of blood, all piglets were euthanized *via* an intravenous injection of sodium pentobarbital (50 mg/kg body weight) (18). A 2 cm jejunal segment was collected and stored in 4% paraformaldehyde for Hematoxylin-eosin (H&E) staining and immunocytochemistry. The jejunal mucosa and cecal chyme were collected, frozen with liquid nitrogen, and then stored at −80 °C for further analysis.

### 2.3 Cell culture and treatment

The IPEC-J2 cells (obtained from the China Center for Type Culture Collection, Wuhan, China) were cultured at 37 °C, 5% CO_2_ in DMEM/F12 (Thermo Scientific, Logan, UT, USA) supplemented with 10% FBS (Gibco, Grand Island, NY, USA) and 1% penicillin-streptomycin (Solarbio, Beijing, China), and the culture medium was replaced every 2 days. After the cell density reached about 80%, either VK (Sigma-Aldrich, MO, USA) or LPS (Sigma-Aldrich, MO, USA) treatment alone or combined treatment for 24 h is carried out. All experiments were conducted at least in triplicate on three separate batches.

### 2.4 Cell viability assay

The effect of VK and LPS on cell viability was assessed using the CCK-8 method. IPEC-J2 cells were cultured in 96-well plates for 24 h, then cultured with VK or LPS at varying levels for 24 h. Moreover, IPEC-J2 cells were treated using another protocol: the cells were inoculated with VK and LPS for 24 h. Then, 10 μL of CCK-8 reagent (Vazyme, Nanjing, China) was added to each well, incubation was continued for 1 h, and the absorbance values at 450 nm were read using a microplate reader (Bio-Rad, Hercules, CA, USA).

### 2.5 Intestinal morphology

The jejunal segments fixed 4% paraformaldehyde solution were dehydrated with a gradient concentration of ethanol, and embedded in paraffin blocks as previously described (16). The paraffin sections were 4 μm thick and then stained with hematoxylin and eosin. The villus height and crypt depth were measured using an automatic digital slice scanning and application system (VM1, Motic China Group Co., Ltd., China).

### 2.6 Serum analysis and cytokine measurement

Serum permeability indicators D-lactate, diamine oxidase (DAO), LPS, reactive oxygen (ROS), and cell supernatant cytokines (TNF-α, IL-1β, IL-6, IL-8) were determined using an ELISA kit (Nanjing Jiancheng Bioengineering Institute, Nanjing, China), according to the manufacturer's instructions.

### 2.7 Real-time PCR analysis

Total RNA was extracted from jejunal tissue or cells using Trizol Reagent following the manufacturer' instructions (Vazyme, Nanjing, China). The quality and concentration were determined using a Nanodrop 2000 (Thermo Fisher Scientific, Waltham, MA, USA). RNA-reverse transcription and quantitative PCR were conducted according to the previous study (12). The primer sequences are displayed in Table 2. *GAPDH* was used the reference gene.

**Table 2:** Primers employed in qPCR.

**Gene**	**Accession number**	**Primer sequences 5^′^ → 3^′^)**
*GAPDH*	XM_047787748.1	F:ACCCAGAAGACTGTGGATGG
		R:AAGCAGGGATGATGTTCTGG
*mucin 1*	XM_021089729.1	F:CAGTCCATGTTGGCACCTCCA
		R:TTTGCTGACTGGGGACATGG
*mucin 2*	XM_021082584.1	F:GGTCGAGTACATCCTGCTGAC
		R:TGTTCCACACGAGAGCAAGG
*mucin 4*	XM_021068272.1	F:TGGGTGTTTCTGAGCTGCTT
		R:AGTTGTCATAGTGTTTCCACCCA
*mucin 13*	NM_001105293.1	F:TTGGCTACAGTGGAGTTGGC
		R:ACGAATGCAATCACCAGGCT
*claudin-1*	NM_001244539.1	F:GGTGACAACATTGTGACGGC
		R:TTACCATCAAGGCACGGGTT
*occludin*	XM_005672525.3	F:TAATGGGCGTCAACCCAACA
		R:GTAGAGTCCAGTCACCGCAG
*ZO-1*	XM_047766890.1	F:GCCATCCACTCCTGCCTAT
		R:CGGGACCTGCTCATAACTTC
*TNF-α*	NM_214022.1	F:TTGAGCATCAACCCTCTGGC
		R:ATTGGCATACCCACTCTGCC
*IL-1β*	NM_001302388.2	F:CCGCCAAGATATAACTGAC
		R:GCAGCAACCATGTACCAA
*IL-6*	NM_214399.1	F:ACCGGTCTTGTGGAGTTTCA
		R:GCATTTGTGGTGGGGTTAGG
*IL-8*	NM_213867.1	F:TTCCAAACTGGCTGTTGCCT
		R:ACAGTGGGGTCCACTCTCAA
		R:ATTGCGACACACTGGAGACC
*GSH-Px*	NM_214201.1	F:TGAATGGCGCAAATGCTCAC
		R:ATTGCGACACACTGGAGACC
*CAT*	XM_021081498.1	F:GCCGCCTATTTGCCTATCCT
		R:TCCCCAGAATAGCGGGTACA
*SOD*	NM_001190422.1	F:GTTGGAGACCTGGGCAATGT
	R:CGGCCAATGATGGAATGGTC
*P38*	XM_003356615.4	F:AGCTTCAGCAGATTATGCGTC
		R:CTCATGGCTTGGCATCCTGT
*ERK1*	XM_021088019.1	F:GATAGTAAAGGGGCAGCCGT
		R:TGATGGCCACTCGAGTCTTG
*NKAP*	NM_001244969.1	F:TTGCAGCAAATCCTCTCGCT
		R:GCCACTCGACTGATGGCTAA
*PI3K*	XM_021102203.1	F:GGACAATTACTGCCACCCCA
		R:TTCCGAAGCTGATTGGGCAT
*AKT*	XM_021081501.1	F:ACCAAGACGACAGCATGGAG
		R:CTGGCCGAGTAGGAGAACTG

### 2.8 Western blot analysis

Western blot analysis was performed as described previously (19). The primary antibodies were used as the following: occludin (proteintech, Cat No. 27260-1-AP), claudin-1 (proteintech, Cat No. 13050-1-AP), TNF-α (proteintech, Cat No. 60291-1-Ig), IL-1β (proteintech, Cat No. 16806-1-AP), IL-6 (proteintech, Cat No. 21865-1-AP), PI3K (ABclonal, Cat No. A4992), p-PI3K (ABclonal, Cat No. AP0427), AKT (proteintech, Cat No. 10176-2-AP), p-AKT (proteintech, Cat No. 66444-1), NF-κB p65 (CST, Cat No. 6956T), p-NF-κB p65 (CST, Cat No. 3033T), p38 (CST, Cat No. 8690T), p-p38 (CST, Cat No. 4511T), ERK (CST, Cat No. 4695T), p-ERK (CST, Cat No. 4370T), β-actin (proteintech, Cat No. 66009-1-Ig), GAPDH (proteintech, Cat No. 60004-1-Ig). Secondary antibodies were purchased from CST (Goat Anti-Mouse IgG, Cat No. 7074P2 and Goat Anti-rabbit IgG, Cat No. 91196S). The band gray value was analyzed by Image J, with β-actin or GAPDH as the internal reference.

### 2.9 Immunofluorescence staining

Tissue sections were deparaffinized, rehydrated, subjected to antigen retrieval, and subsequently blocked with 5% bovine serum albumin. Then, tissue sections were incubated with the primary antibody (ZO-1, occludin, claudin-1) at 4 °C overnight, followed by staining with Cy3 conjugated Goat Anti-Rabbit IgG (H + L) secondary antibody at room temperature for 50 min. Cell nucleus was stained with 4′,6-diamidino-2-phenylindole (DAPI), and the images were captured using a fluorescence microscope.

### 2.10 RNA-Seq analysis

Total RNA was extracted from the cells in the CON, LPS, and LPS+VK groups. The concentrations and quality of extracted RNA were checked by Nandrop 2000 and Agilent 2000 Bioanalyzer (Agilent, CA, USA). The obtained mRNA was reverse transcribed into double-stranded cDNA, purified for cDNA library construction, and finally sequenced by Guangzhou Gidi Bio-Tech Co., Ltd. (Guangzhou, China). Briefly, the raw sequencing data was filtered by adapter removal and low-quality filtering. The trimmed reads were aligned to the *Sus scrofa* reference genome using HISAT2, and then StringTie was used for reference genome-guided transcriptome assembly and gene expression quantification. Differentially expressed genes (DEGs) were identified using DEseq2 and genes with adjusted log2|fold-change| values and *P* < 0.05 were defined as DEGs. Based on GO and KEGG pathway categories, ClusterProfiler was used to conduct functional enrichment analysis for the significantly annotated DEGs and potential genes in identified modules. *P* < 0.05 was considered significant. Gene set enrichment analysis (GSEA) was conducted using the functions in the ClusterProfiler package, and the gene list was sorted by log2 fold-change.

### 2.11 Proteomic analysis

Protein extraction and digestion were done according to the previous study (20). LC-MS/MS analysis was conducted using a timsTOF Pro2 (Bruker Daltonics, MA, USA) coupled to UltiMate 3000 (Thermo Fisher Scientific, MA, USA). Approximately 200 ng of the sample was loaded onto a AUR3-15075C18 analytical column (IonOpticks, Fitzroy, Australia) and separated using a 60-min gradient at a column temperature of 50 °C. The column flow rate was controlled at 400 nL/min. The gradient started from 4% B phase (80% acetonitrile, 0.1% formic acid) and increased to 28% within 25 min, then rose to 44% within 10 min, increased to 90% within another 10 min, and remained at 90% for 7 min, with 4% returning to equilibrium after 8 min. The mass spectrometer uses the diaPASEF mode for DIA data acquisition, with the scanning range set from 349 to 1,229 m/z, and the isolation window width is set at 40 Da. During the PASEF MSMS scan process, the collision energy increases linearly with the ion mobility, rising from 59 eV (1/K0 = 1.6 Vs/cm^2^) to 20 eV (1/K0 = 0.6 Vs/cm^2^). The MS raw data for each sample was analyzed using Spectronaut18 default parameters for identification and quantitation analysis. Data analysis was performed on the online platform of Kidio Bio Cloud Platform (https://www.omicsmart.com/). Moreover, the integrated analysis of transcriptome and proteome was also performed on the online platform of Kidio Bio Cloud Platform.

### 2.12 Gut microbiota analysis

16S rRNA gene sequencing and bioinformatics analysis were performed as described previously (21). Bacterial V3-V4 region primer 341F (5′-CCTACGGGNGGCWGCAG-3′) and 806R (5′-GGACTACHVGGGTWTCTAAT-3′) were used for the PCR amplification of the target region.

### 2.13 Statistical analysis

All data in this study were statistically analyzed by using the SPSS software (Version 29.0), while graphs were generated using GraphPad Prism 9 software (GraphPad Software, La Jolla, USA). Data analysis employed the single-factor variance method, and pairwise comparisons were conducted using the *t*-test. Data are presented as the mean ± standard error of the mean (SEM) and *P* < 0.05 was considered statistically significant.

## 3 Results

### 3.1 VK ameliorated intestinal barrier injury in LPS-induced intestinal injury of piglets

**Figure 1:**
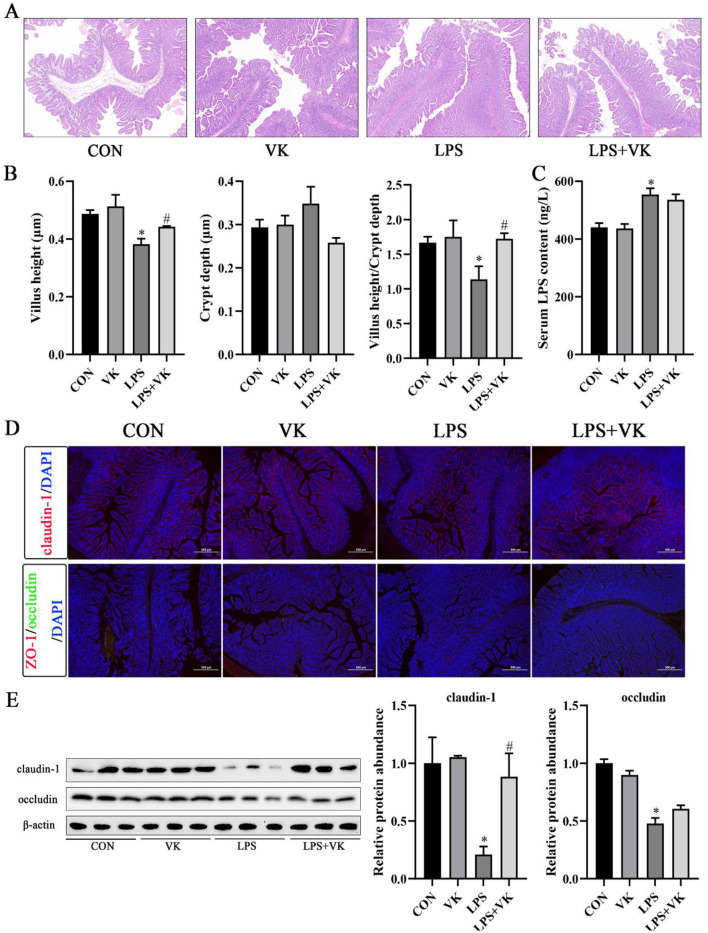
Effects of VK supplementation on intestinal morphology and tight junctions in LPS-induced intestinal injury of piglets. **(A)** HE staining of piglet jejunum. Scale bar, 200 μm; **(B)** Jejunal villus height, jejunal crypt depth, and ratio of villus height to crypt depth in piglets; **(C)** Serum LPS concentration in piglets; **(D)** Immunofluorescence detected the expression of claudin-1, ZO-1, and occludin in piglets (scale bar represents 500 μm); **(E)** Relative protein levels of claudin-1 and occludin in the jejunum. All data are expressed as the mean ± SEM. *P < 0.05 as compared to the CON group, ^#^*P* < 0.05 as compared to the LPS group.

There were no differences in ADG, ADFI, and F/G of piglets among the four groups (Table 3). The results of the jejunal morphology showed that the LPS group exhibited lower villus height and ratio of villus height to crypt depth compared to the CON group, whereas VK supplementation alleviated these changes (*P* < 0.05; Figures 1A, B). LPS adminstration significantly increased the serum LPS concentration in piglets (Figure 1C). The concentration of DAO, which was substantially increased by LPS administration, was notably restored following VK supplementation (*P* < 0.05; Supplementary Figure S1A). Compared with the CON group, LPS administration significantly the protein expression of claudin-1 and occludin (*P* < 0.05). Compared with the LPS group, the LPS+VK group exhibited a significant increase in occludin and claduin-1 protein expression (*P* < 0.05; Figures 1D, E, Supplementary Figure S1B). Besides, the results of gene expression showed that LPS administration decreased *occludin, claudin-1*, and *mucin 13* gene expression (*P* < 0.05). In comparison to the LPS group, VK supplementation significantly increased *occludin, claudin-1*, and *mucin 13* gene expression (*P* < 0.05; Supplementary Figure S1C). These results were mirrored at the IPEC-J2 cells, where similar changes were observed in LPS-challenged IPEC-J2 cells (Supplementary Figure S2D).

**Table 3:** Effects of VK supplementation on growth performance in LPS-induced intestinal injury of piglets.

**Item**	**CON**	**VK**	**LPS**	**LPS+VK**	**SEM**	***P-*value**
Initial body weight, kg	7.55	7.53	7.54	7.52	0.10	1.00
Final body weight, kg	12.99	13.34	12.93	13.31	0.33	0.96
ADG, g	259.13	276.59	256.35	275.79	13.02	0.93
ADFI, g	482.87	481.94	497.69	476.85	13.29	0.96
F/G	1.90	1.80	1.98	1.78	0.05	0.39

All data are expressed as the mean ± SEM.

ADG, average daily gain; ADFI, average daily feed intake; F:G, average daily feed intake/average daily gain.

### 3.2 VK ameliorated inflammatory factors expression in LPS-induced intestinal injury of piglets

**Figure 2:**
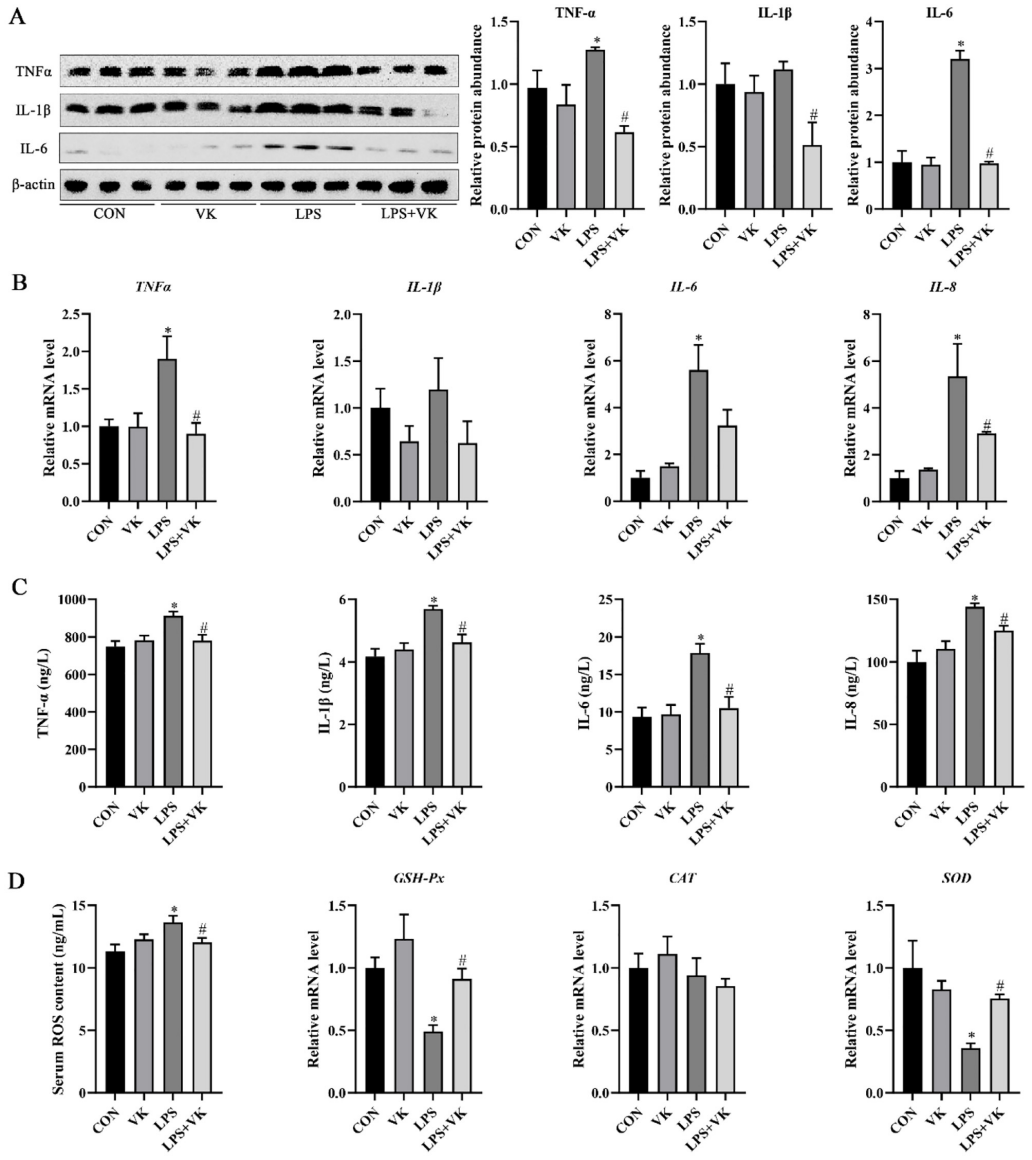
Effects of VK supplementation on inflammatory markers in LPS-induced intestinal injury of piglets and LPS-challenged IPEC-J2 cells. **(A)** Relative protein levels of TNF-α, IL-1β, and IL-6 in the jejunum; **(B)** Relative gene expression levels of *TNF-*α, *IL-1*β, *IL-6*, and *IL-8* in the jejunum; **(C)** levels of TNF-α, IL-1β, IL-6, and IL-8 in LPS-challenged IPEC-J2 cells; **(D)** Serum ROS concentration and relative gene expression levels of GSH-Px, CAT, and SOD. All data are expressed as the mean ± SEM. *P < 0.05 as compared to the CON group, ^#^*P* < 0.05 as compared to the LPS group.

As shown in Figure 2A, LPS significantly promoted the protein expression of the inflammatory indicators TNF-α and IL-6 in the jejunum (*P* < 0.05). Supplemented with VK, however, significantly decreased the protein expression of TNF-α, IL-1β, and IL-6 (*P* < 0.05). Additionally, LPS-induced damage resulted in a noteworthy increase in the mRNA of *TNF-*α, *IL-6*, and *IL-8* (*P* < 0.05). However, VK supplementation effectively restored *TNF-*α and *IL-8* expression (*P* < 0.05; Figure 2B). Furthermore, these results were verified in LPS-challenged IPEC-J2 cells (Figure 2C). The concentration of serum ROS which was substantially increased by LPS administration, was notably restored following VK supplementation. Moreover, LPS administration reduced the mRNA levels of *GSH-Px* and *SOD* in the jejunum, while VK supplementation reversed these changes (*P* < 0.05; Figure 2D).

### 3.3 Transcriptomic and proteomic analysis of the intestinal protective effects of VK

**Figure 3:**
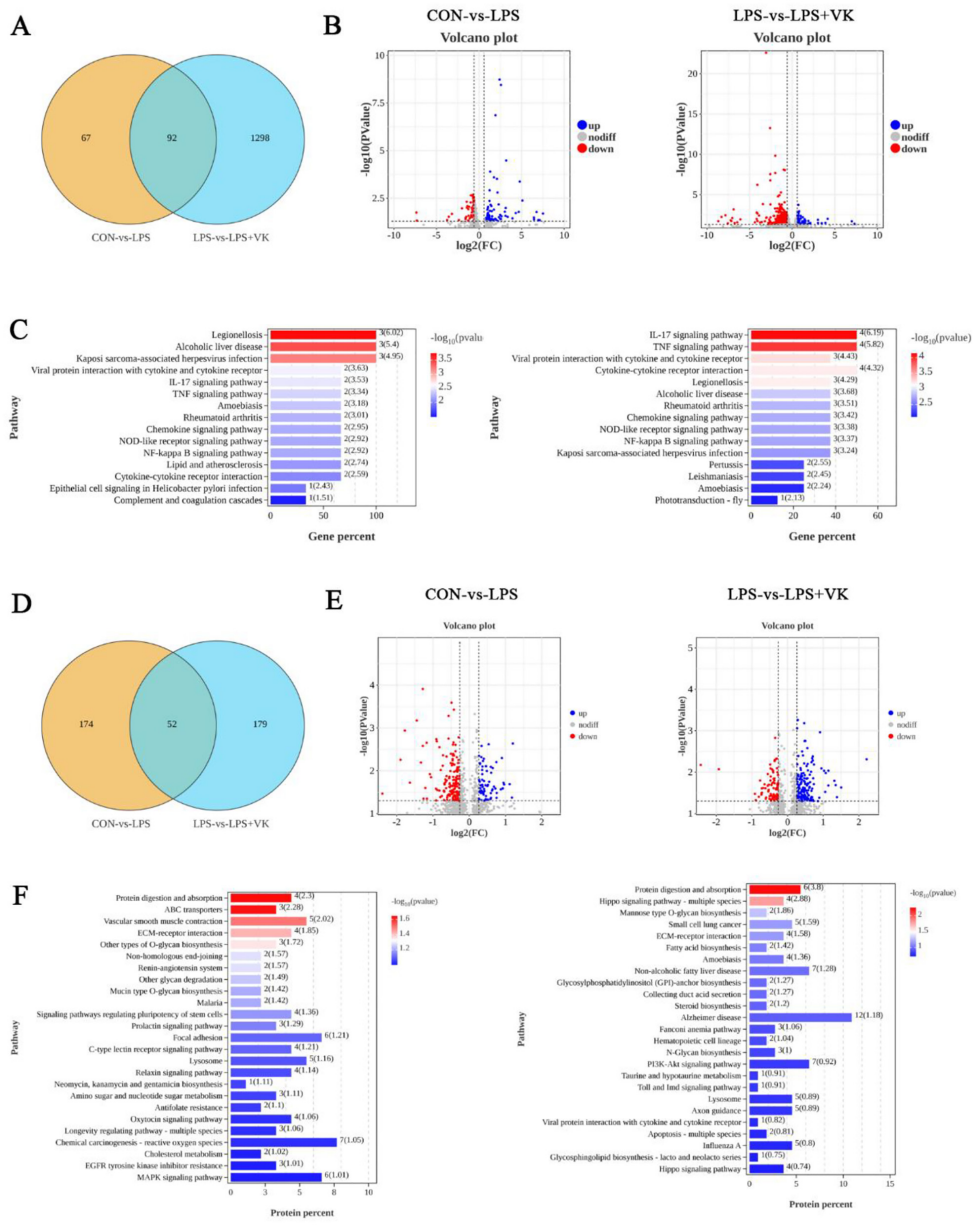
Transcriptomic data and proteomic data in LPS-challenged IPEC-J2 cells. **(A)** Venn diagram of transcriptomic data; **(B)** Volcano plot of gene expression from transcriptomic data; **(C)** KEGG enrichment of DEGs in various pathways between the CON and LPS groups, as well as between the LPS and LPS+VK groups from the transcriptomic data; **(D)** Venn diagram of proteomic data; **(E)** Volcano plot of protein expression from proteomic data; **(F)** KEGG enrichment of DEGs in various pathways between the CON and LPS groups, as well as between the LPS and LPS+VK groups from the proteomic data.

To further reveal the potential protective mechanism of VK on intestinal inflammation, transcriptomic and proteomic analysis were employed to examine the KEGG and GO enrichment pathways. One hundred fifty-nine DEGs were analyzed between the CON and LPS groups, with 97 genes upregulated and 62 genes downregulated. In contrast, 1,390 DEGs screened between the LPS and LPS+VK groups, comprising 87 upregulated and 1,303 downregulated genes (Figures 3A, B). The KEGG pathway enrichment analysis showed that DEGs in the CON vs. LPS and LPS vs. LPS+VK comparisons were associated with key signaling pathways (Figure 3C). Notably, 15 pathways were prominently annotated in the LPS group compared with the CON group, including the IL-17, TNF, and NF-κB signaling pathways. Similarly, the LPS+VK group exhibited enrichment in several of these same pathways. Moreover, GO enrichment analysis of DEGs revealed their biological significance, highlighting multiple functional categories (Supplementary Figures S3A, B).

Two hundred twenty-six proteins were analyzed between the CON and LPS groups, with 71 proteins upregulated and 155 proteins downregulated. In contrast, 231 proteins screened between the LPS and LPS+VK groups, comprising 148 upregulated and 83 downregulated proteins (Figures 3D, E). The KEGG pathway enrichment analysis indicated that 25 pathways were prominently annotated in the LPS group compared with the CON group, including the MAPK signaling pathway. However, the LPS+VK group exhibited enrichment in the PI3K-AKT signaling pathway (Figure 3F). The enrichment of DEGs and proteins underscored the complexity of the protective mechanisms in which VK mitigated intestinal inflammation induced by LPS.

### 3.4 VK ameliorated intestinal inflammation by inhibiting MAPK and PI3K-AKT signaling pathways

**Figure 4:**
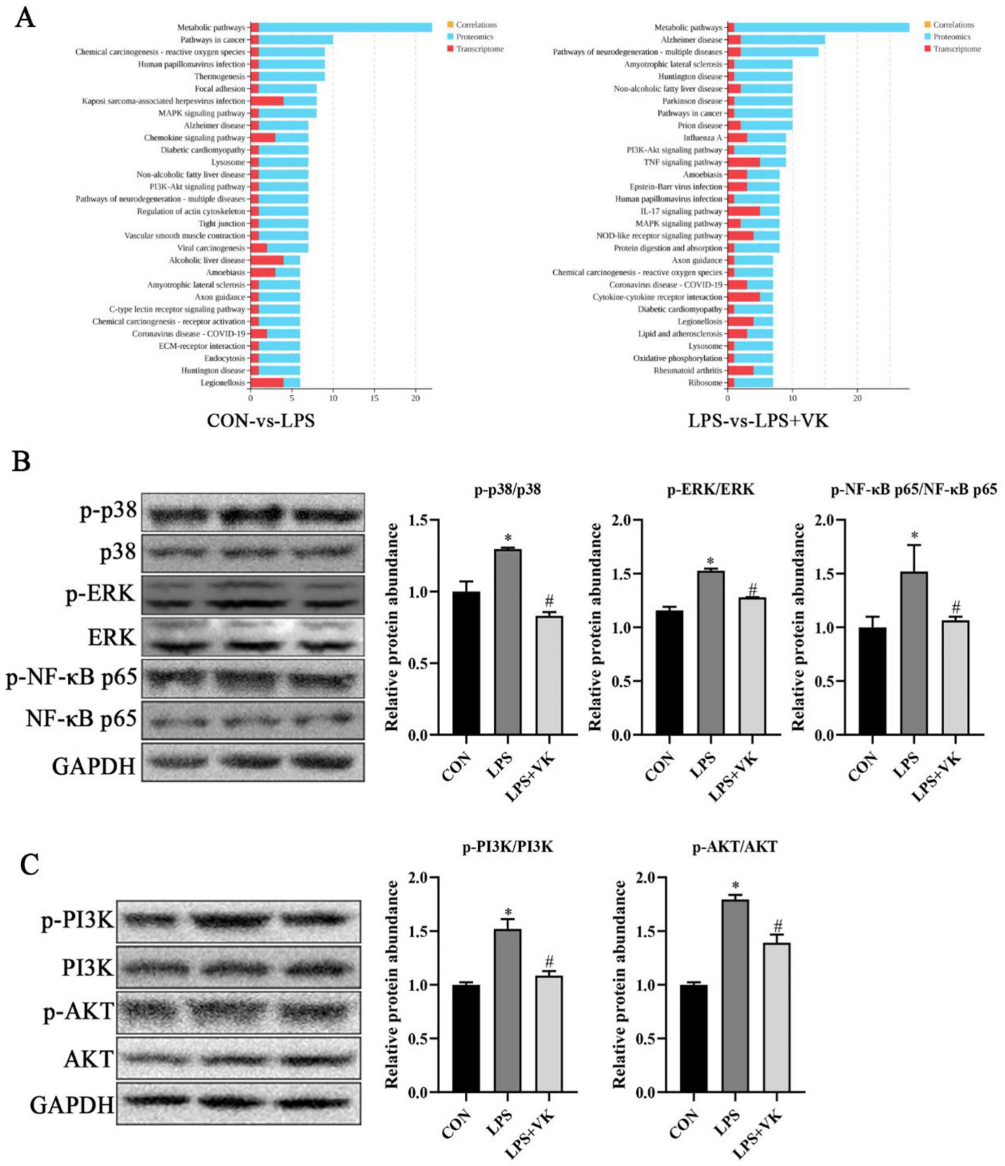
Effects of VK supplementation on related pathways in LPS-challenged IPEC-J2 cells. **(A)** KEGG enrichment of DEGs and proteins in various pathways between the CON and LPS groups, as well as between the LPS and LPS+VK groups from transcriptome and proteome combined analysis; **(B)** Relative protein levels of p-p38, p38, p-ERK, ERK, p-NF-κB p65, and NF-κB p65; **(C)** Relative protein levels of p-PI3K, PI3K, p-AKT, and AKT. All data are expressed as the mean ± SEM. *P < 0.05 as compared to the CON group, ^#^*P* < 0.05 as compared to the LPS group.

Transcriptomic and proteomic combined analysis highlighted the role of the MAPK and PI3K-AKT signaling pathways in VK alleviating LPS-induced intestinal inflammation (Figure 4A). To verify this hypothesis, we analyzed the expression levels of key proteins in the MAPK and PI3K-AKT pathways. Results showed that LPS administration led to an obvious increase in the mRNA levels of *p-38, ERK-1, NKAP, PI3K*, and *AKT* (Supplementary Figure S4), as well as the phosphorylation levels of p-p38, p-ERK, p-NF-κB p65, p-PI3K, and p-AKT, while VK supplementation significantly reversed these changes (Figures 4B, **C**). Moreover, the levels of nonphosphorylated proteins of p38, ERK, NF-κB p65, PI3K, and AKT remained largely unchanged. These findings demonstrated that the underlying mechanism of VK against LPS-induced intestinal dysfunction was primarily mediated through attenuation of the excessive activation of the MAPK and PI3K-AKT signaling pathways.

### 3.5 VK protected against disruption of gut microbiota in LPS-induced intestinal injury of piglets

**Figure 5:**
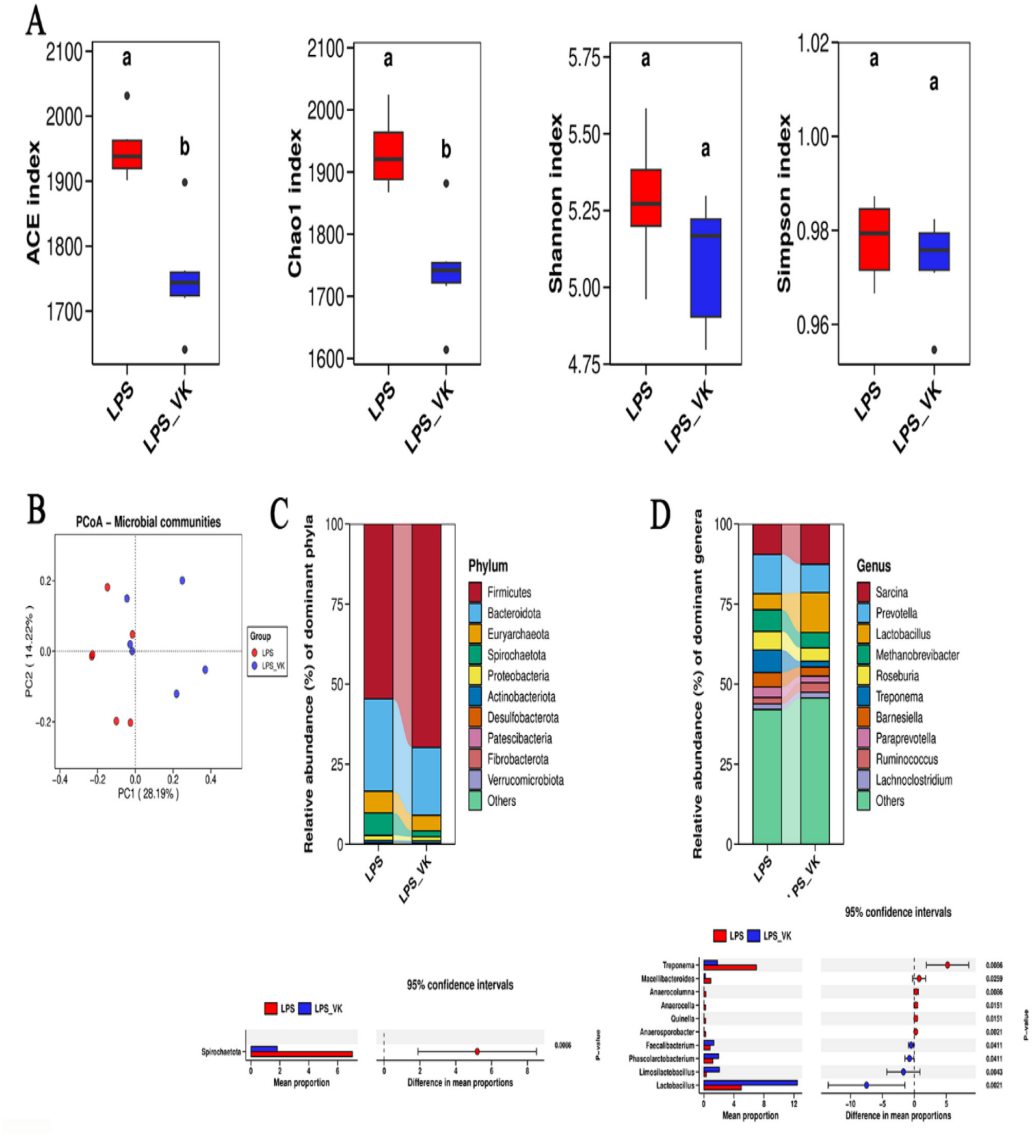
Effects of VK supplementation on gut microflora in LPS-induced intestinal injury of piglets. **(A)** Alpha diversity index of ACE, Chao 1, Shannon, and Simpson; **(B)** Principal component analysis; **(C)** Abundance of the intestinal microbiota at the phylum levels; **(D)** Abundance of the intestinal microbiota at the genus levels. All data are expressed as the mean ± SEM. ^a, b^values with different superscripts mean significant difference (*P* < 0.05).

The effects of VK on gut microbiota was investigated by using 16S rRNA sequencing. As shown in Figure 5A, various α-diversity indices reflected species richness and evenness across the groups. Supplemented with VK decreased the ACE and Chao1 indexes compared with the LPS group (*P* < 0.05). PCoA analysis showed that a distinct separation among the LPS and LPS+VK groups (Figure 5B). Figure 5C shows the relative abundance of the intestinal flora at the phylum level. Compared with the LPS group, Spirochaetato showed a significant decrease in the LPS+VK group (*P* < 0.05). Furthermore, VK also influenced the intestinal flora at the genus levels (Figure 5D), increasing *Faecalibacterium, Phascolarctobacterium, Limosilactobacillus*, and *Lactobacillus* while reducing *Treponema, Macellibacteroides, Anaerocolumna, Anaerocella, Quinella*, and *Anaerosporobacter* levels (*P* < 0.05).

## 4 Discussion

Early weaning induces weaning stress in piglets, leading to gut microbiota dysbiosis and mucosal barrier dysfunction, which manifests as reduced physical condition, severe diarrhea, compromised immunity, and systemic inflammatory response (22, 23). Therefore, reducing intestinal mucosal barrier damage and inflammation while maintaining a healthy gut microbiota during the weaning period is crucial for ensuring swine production, animal welfare, and consumer safety. LPS, also as known endotoxin, binds to the TLR4 receptor on host cells to initiate intracellular signaling pathways, leading to the production and release of inflammatory cytokines, and is commonly administered via intraperitoneal injection to establish intestinal injury models in weaning piglets (24–26). VK, a naphthoquinone compound with an isoprene-type side chain, plays a critical role in regulating the immune responses, oxidative stress, and intestinal health, among other functions (11, 12, 27). However, its potential mechanism of operation on intestinal health still remains unknown. Therefore, we established the LPS-induced piglet and cell models to explore the protective effects of VK on relieving inflammatory response.

Consistent with the previous study (28), our results indicated that LPS disrupted the morphological structure of the piglet jejunum, mainly manifested as a decrease in villus length and the ratio of villus height to crypt depth. Serum D-lactate and DAO can be used as indicators of intestinal integrity, as they are usually present in extremely low concentrations in the bloodstream, but their levels will significantly increase when the mucosa is damaged (29). The present study showed that the LPS challenge led to a significant increase in LPS and DAO levels. These biological phenotypic phenomena demonstrated that we have successfully established an experimental model of intestinal inflammation in piglets (30). However, VK supplementation effectively maintained jejunal villi morphology and reduced the DAO level in LPS-challenged piglets, which indicated that VK may have protective effects against intestinal barrier injury in response to LPS stimulation. Tight junction proteins, such as occludin, claudin-1, and ZO-1, prevent intestinal inflammation by separating the internal and external environments and blocking the passage of potentially harmful substances (31, 32). VK supplementation restored the LPS-induced reduction in these tight junction proteins, thus improving the intestinal barrier function in piglets. Consistent with this, our earlier research also confirmed that VK supplementation elevated occludin, claudin-1, and ZO-1 expression in colitis mice (12). Taken together, VK supplementation has a protective effect on the intestinal integrity and barrier function of piglets.

Although environmental factors, feed, bacteria, and viruses induce intestinal inflammation through distinct mechanisms, a shared feature is that once initiated, the inflammation becomes amplified, resulting in the production and release of large amounts of inflammatory cytokines that cause severe damage to the intestinal tract (33). As it has been known, the TNF-α, IL-1β, IL-6, and IL-8 are typical pro-inflammatory cytokines (34). In the present study, the LPS administration promoted the expression and secretion of pro-inflammatory cytokines such as TNF-α, IL-1β, IL-6, and IL-8 in both the jejunum and IPEC-J2 cells compared to the CON group. In contrast, VK supplementation reduced the expression of these pro-inflammatory cytokines, thereby improving the intestinal inflammation in piglets. In agreement with the present study, Wang et al. (12) and Shiraishi et al. (35) demonstrated that VK exerts a protective effect against DSS colitis by down-regulating TNF-α, IL-1β, and IL-6 and up-regulating IL-10. Furthermore, oxidative stress plays a crucial role in damaging the intestinal barrier function and causing intestinal diseases (36). In this study, VK supplementation enhanced the antioxidant capacity of LPS-challenged piglet by reducing the ROS concentration and increasing the mRNA expression of *GSH-Px* and *SOD*. These results aligned with the previous study that mitigated oxidative stress by enhancing antioxidant capacity in the intestine of juvenile Jian carp (11).

Transcriptomic analysis is employed to explore gene expression differences under specific conditions through high-throughput sequencing technology, thereby identifying key regulatory genes, revealing their functional mechanisms, and elucidating the molecular regulatory networks of biological processes (37). The proteomic analysis systematically examines protein dynamics in biological samples, revealing the mechanisms underlying life activities (38). In this study, VK modulated DEGs and proteins through intricate physiological and biochemical processes. KEGG enrichment results of the combined analysis of transcriptome and proteomic analysis indicated that the DEGs and proteins in the VK-treated group were predominantly associated with some crucial pathways, such as MAPK and PI3K-AKT. Previous study has demonstrated that VK has the potential to alleviate LPS-induced inflammation by inhibiting NF-κB and IKKα/β phosphorylation (13). Additionally, VK has been proven to suppress pyroptosis by restraining the NF-κB and JNK pathways in THP-1 Cells (39). MAPK is an upstream target of the NF-κB signaling pathway, and its key components include ERK and p38 (40). Zhang et al. proved that low-molecular-weight chitosan attenuated LPS-induced inflammation by suppressing the NF-κB pathway in IPEC-J2 cells (41). Furthermore, chalcone could ameliorated DSS-induced colitis by inhibiting ERK and p38 phosphorylation levels in mice (42). Consistent with these findings, key proteins within the MAPK pathway, including p-p38, p-ERK, and p-NF-κB p65, were obviously elevated by LPS, but VK supplementation effectively inhibited their phosphorylation levels in the present study. A recent study has revealed that the activation of the PI3K-Akt signaling pathway is involved in the development process of ulcerative colitis (43). Furthermore, another study has found that inhibiting the phosphorylation of proteins in the PI3K-AKT signaling pathway can produce a significant anti-inflammatory effect in the piglets (19). These evidences suggest that inhibiting the PI3K-Akt signaling pathway can be regarded as a novel therapeutic strategy for intestinal inflammation. In the present study, LPS administration increased the mRNA levels of *PI3K* and *AKT*, as well as the protein levels of p-PI3K and p-AKT, while VK supplementation effectively inhibited their expression. Collectively, the anti-inflammatory role of VK was mainly attributed to the modulation in the excessive activation of the MAPK and PI3K-AKT signaling pathways induced by LPS.

Gut microbiota are crucial for maintaining health and play a significant role in the pathogenesis of gastrointestinal diseases, making them an integral component of the host organism (44). A stable gut microbiota serves as a vital barrier against pathogen invasion and disruption of this balance can lead to various diseases, such as infectious diarrhea, inflammatory bowel disease, irritable bowel syndrome, etc. (45). *Spirochaetota* can cause both acute and chronic inflammation through mechanisms such as the direct release of virulence factors, the deposition of immune complexes, and molecular mimicry, which affects multiple organ systems (46, 47). VK supplementation reduced the abundance of *Spirochaetota* in the LPS-induced piglets. *Faecalibacterium, Phascolarctobacterium, Limosilactobacillus*, and *Lactobacillus* are beneficial bacteria in the gut. *Faecalibacterium* is linked to various health tissues, including colorectal cancer, dermatitis, and depression (48). *Phascolarctobacterium* promotes the production of short-chain fatty acids, modulating the innate immune system in mice and thereby alleviating obesity and metabolic disorders (49). *Limosilactobacillus* and *Lactobacillus* are almost naturally present in the intestines of all vertebrates and mammals, and it can regulate the body's immunity (50, 51). LPS-induced inflammation in this study resulted in a reduction of these bacteria, which was successfully mitigated by VK supplementation, preserving these beneficial bacteria. *Treponema*, a harmful bacteria in the gut, has been reported to be associated with the occurrence of periodontal disease (52). VK supplementation effectively regulated the abundance of *Treponema*, thus promoting the microbial balance within the gut.

## 5 Conclusion

In conclusion, VK effectively suppressed LPS-induced intestinal inflammation, restored intestinal integrity, and enhanced barrier function. The anti-inflammatory mechanisms of VK were associated with the inhibition of MAPK and PI3K-AKT signaling pathways as well as the modulation in the gut microbiota. These findings highlight the potential of VK as a natural feed additive to alleviate intestinal dysfunction.

## Data Availability

The original contributions presented in the study are publicly available. This data can be found here: https://www.ncbi.nlm.nih.gov/sra/PRJNA1348319.
