# Supplementary material for: Vitamin K protects against lipopolysaccharide-induced intestinal inflammation in piglets by inhibiting the MAPK and PI3K-AKT pathways
Source: Front Nutr. 2025 Nov 17;12:1704168. doi: 10.3389/fnut.2025.1704168 (PMC12665530; doi:10.3389/fnut.2025.1704168)
Supplement: Supplementary file 1 [file Image_1.pdf]

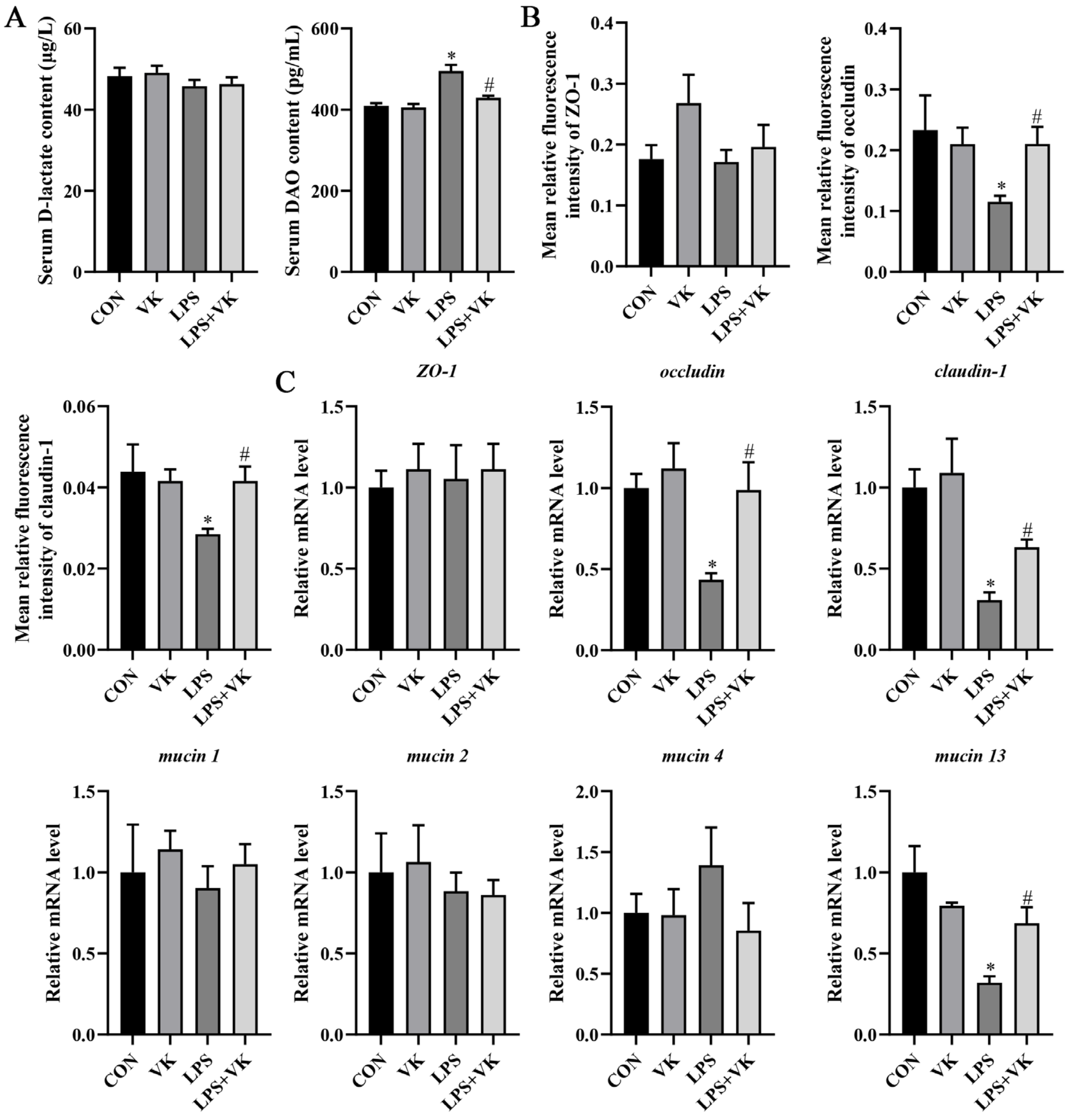

Figure S1 Effects of VK supplementation on indicators related to intestinal barrier function in LPS-induced intestinal injury of piglets. A, Levels of D-lactate and DAO in the serum; B, Quantitative results of immunofluorescence for claudin-1, ZO-1 and occludin in piglets; C, Relative gene expression levels of *ZO-1*, *occludin*, *claudin-1*, *mucin 1*, *mucin 2*, *mucin 4*, and *mucin 13*. All data are expressed as the mean ± SEM. * $P < 0.05$ as compared to the CON group, # $P < 0.05$ as compared to the LPS group.

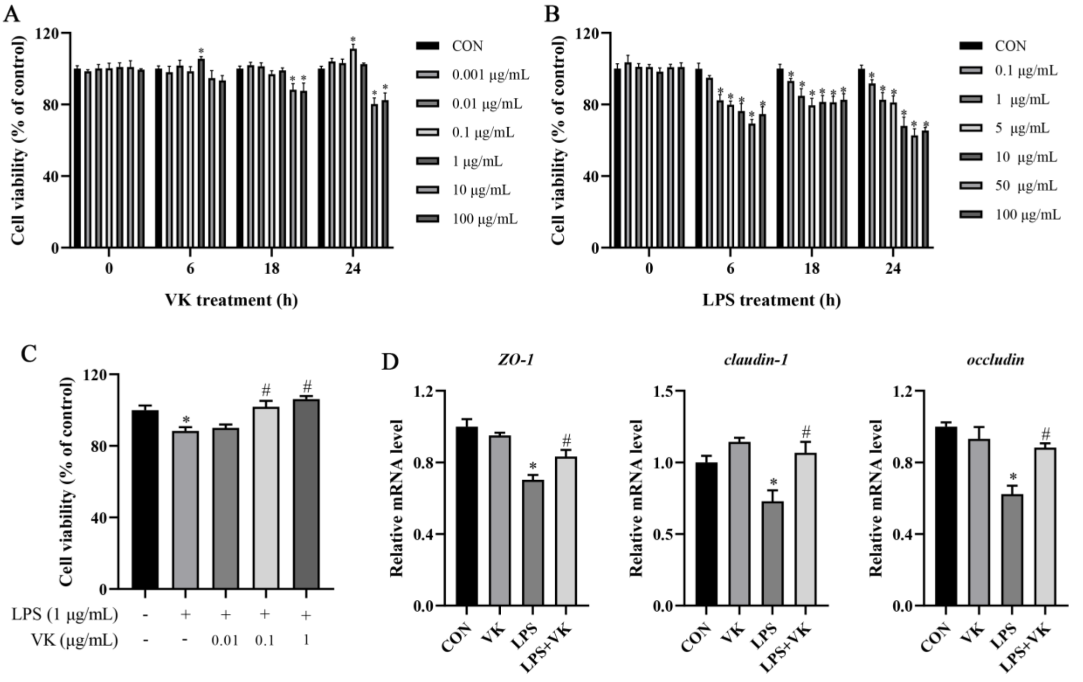

Figure S2 Effects of VK supplementation on viability and expressions of tight junction in LPS-challenged IPEC-J2 cells. A, IPEC-J2 cells were treated with different concentrations of VK; B, IPEC-J2 cells were treated with different concentrations of LPS; C, IPEC-J2 cells challenged with or without LPS (1 μg/mL) were treated with different concentrations with VK; D, Relative gene expression levels of *ZO-1*, *claudin-1*, and *occludin*. All data are expressed as the mean ± SEM. * $P < 0.05$ as compared to the CON group, # $P < 0.05$ as compared to the LPS group.

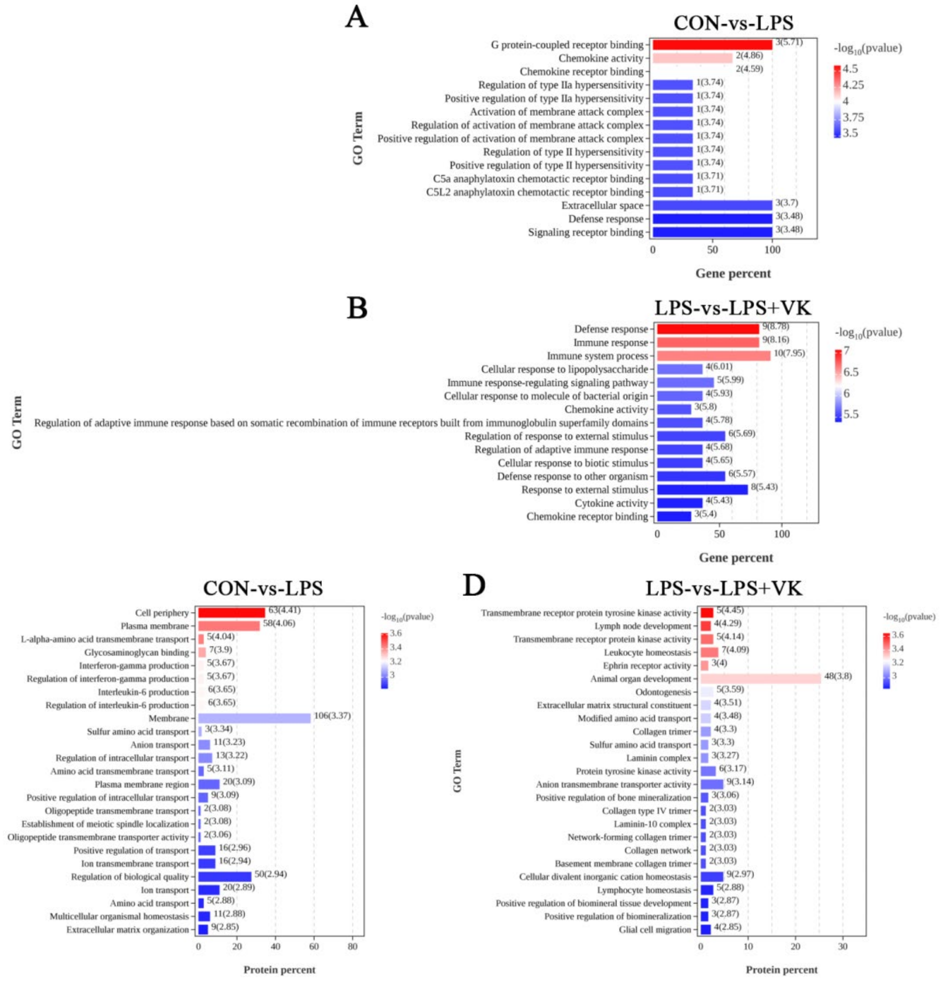

Figure S3 Transcriptomic data and proteomic data in LPS-challenged IPEC-J2 cells. A and B, GO enrichment of DEGs between the CON and LPS groups, as well as between the LPS and LPS+VK groups from the transcriptomic data; C and D, GO enrichment of DEGs between the CON and LPS groups, as well as between the LPS and LPS+VK groups from the proteomic data. N=3.

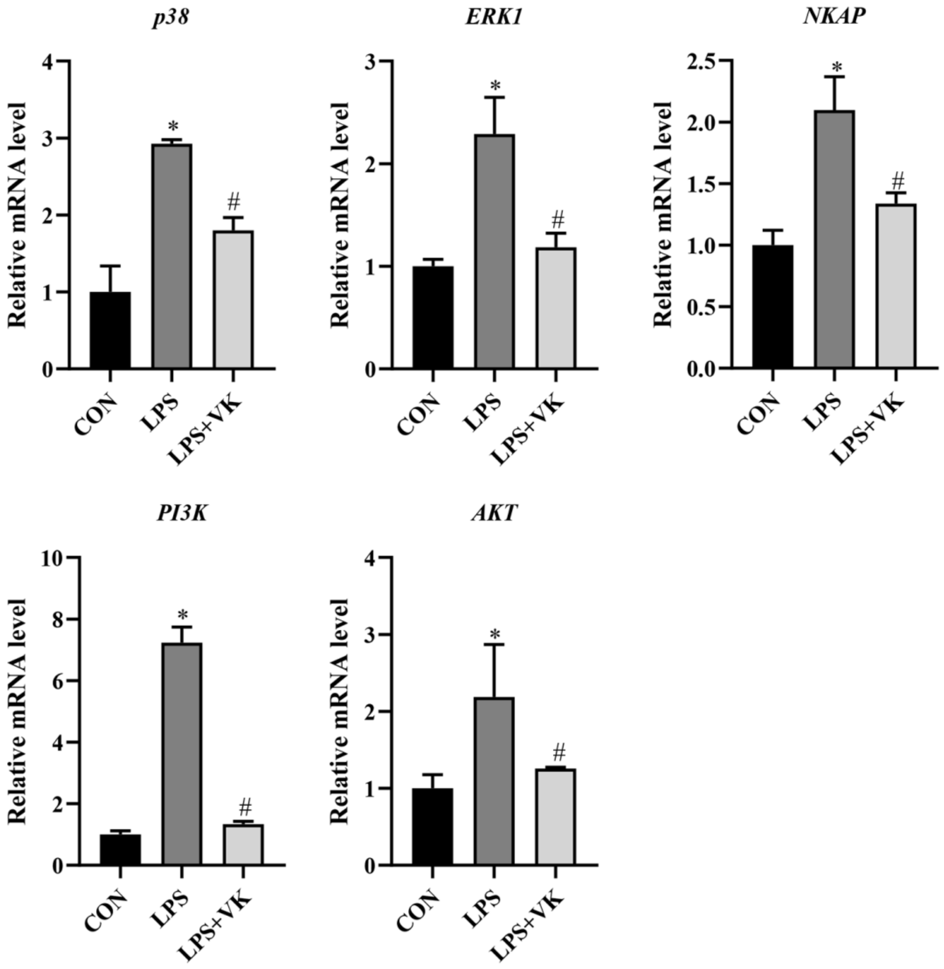

Figure S4 Relative gene expression levels of *p-38*, *ERK-1*, *NKAP*, *PI3K*, and *AKT*. All data are expressed as the mean ± SEM. * $P < 0.05$ as compared to the CON group, $^{\#}$ $P < 0.05$ as compared to the LPS group.
